# Differential contribution of p300 and CBP to regulatory element acetylation in mESCs

**DOI:** 10.1186/s12860-020-00296-9

**Published:** 2020-07-20

**Authors:** Sara Martire, Jennifer Nguyen, Aishwarya Sundaresan, Laura A. Banaszynski

**Affiliations:** Cecil H. and Ida Green Center for Reproductive Biology Sciences, Department of Obstetrics and Gynecology, Children’s Medical Center Research Institute, Hamon Center for Regenerative Science and Medicine, University of Texas Southwestern Medical Center, Dallas, TX 75390 USA

**Keywords:** Enhancer, Chromatin, Acetyltransferase, Embryonic stem cell

## Abstract

**Background:**

The transcription coactivators CREB binding protein (CBP) and p300 are highly homologous acetyltransferases that mediate histone 3 lysine 27 acetylation (H3K27ac) at regulatory elements such as enhancers and promoters. Although in most cases, CBP and p300 are considered to be functionally identical, both proteins are indispensable for development and there is evidence of tissue-specific nonredundancy. However, characterization of chromatin and transcription states regulated by each protein is lacking.

**Results:**

In this study we analyze the individual contribution of p300 and CBP to the H3K27ac landscape, chromatin accessibility, and transcription in mouse embryonic stem cells (mESC). We demonstrate that p300 is the predominant H3K27 acetyltransferase in mESCs and that loss of acetylation in p300KD mESCs is more pronounced at enhancers compared to promoters. While loss of either CBP or p300 has little effect on the open state of chromatin, we observe that distinct gene sets are transcriptionally dysregulated upon depletion of p300 or CBP. Transcriptional dysregulation is generally correlated with dysregulation of promoter acetylation upon depletion of p300 (but not CBP) and appears to be relatively independent of dysregulated enhancer acetylation. Interestingly, both our transcriptional and genomic analyses demonstrate that targets of the p53 pathway are stabilized upon depletion of p300, suggesting an unappreciated view of the relationship between p300 and p53 in mESCs.

**Conclusions:**

This genomic study sheds light on distinct functions of two important transcriptional regulators in the context of a developmentally relevant cell type. Given the links to both developmental disorders and cancer, we believe that our study may promote new ways of thinking about how these proteins function in settings that lead to disease.

## Background

Regulatory elements are defined by an open chromatin state and the binding of transcription factors, which in turn contributes to the recruitment of transcription coactivators such as CBP and p300 [1, 2]. In addition to facilitating specific protein interactions at regulatory elements, CBP and p300 acetylate both histone and nonhistone proteins and are specifically responsible for histone acetylation on H3K27 [2–6]. Traditionally, CBP and p300 are considered to function identically. They share a high degree of sequence identity in structured domains, including their enzymatic HAT domains and their bromodomains [3, 7]. However, CBP and p300 exhibit much lower homology outside of predicted domains and have been reported to interact with different protein partners [2]. A recent study also reports differences in substrate specificity and selectivity based on enzyme levels [8]. Further, CBP and p300 are each required for mammalian development [9, 10] and certain cell types have been shown to be more tolerant of CBP or p300 loss compared to the whole organism [11–14], suggesting that CBP and p300 function nonredundantly in vivo. Although several studies assess differences between CBP and p300 in vitro and at the level of cellular phenotype [8–18], the distinct roles of p300 and CBP in establishing functional chromatin states at a genome-wide level has not been directly explored.

In this study, we analyzed the individual contribution of p300 and CBP to the H3K27ac landscape, chromatin accessibility, and transcription in mESC. To study this question, we performed a series of genomic analyses using isogenic wild-type and p300 knockdown and CBP knockdown stable lines. Overall, we find that p300, and not CBP, is responsible for maintaining H3K27ac at specific regions of the genome in mESCs. p300-mediated acetylation of promoters correlates more strongly with transcription than enhancer acetylation, and changes in acetylation do not correlate with changes in DNA accessibility. Finally, we find that regions that maintain H3K27ac after p300 depletion are enriched with p53 binding motifs, suggesting an unappreciated view of the relationship between p300 activity and p53 in mESCs.

## Results

### p300 maintains enhancer acetylation in mESCs

**Fig. 1:**
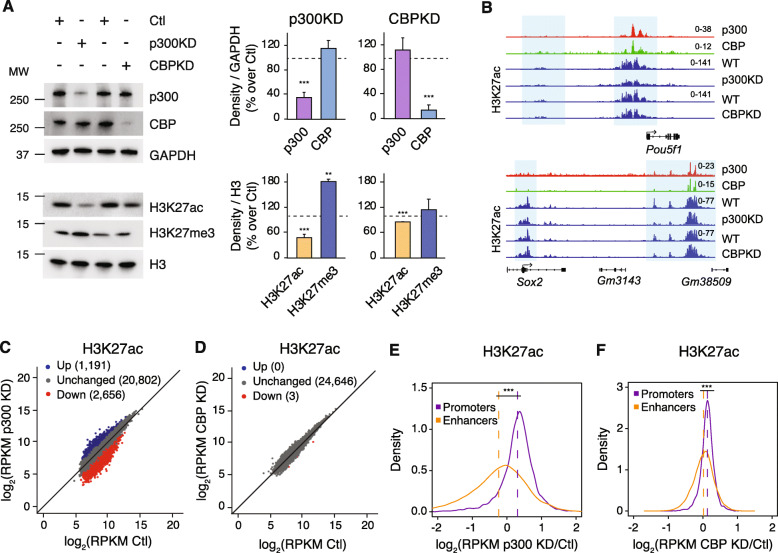
p300 Maintains Enhancer Acetylation in mESCs. **a** Immunoblot of whole cell lysates from mESCs transfected with scramble shRNA or either p300 or CBP shRNA and relative quantification. Data are represented as mean ± S.D. of at least 3 independent experiments. *** *p* < 0.001 vs Ctl. **b** Browser track of p300 [19] and CBP [20] in WT mESC and H3K27ac in Control and P300KD or CBPKD mESCs. Promoter and enhancer regions for two important pluripotency transcription factors (*Pou5f1* and *Sox2*) are highlighted in blue. **c**,**d** H3K27ac ChIP-seq enrichment in mESCs expressing scramble control (Ctl) shRNA compared with **b** p300 shRNA (p300 KD) or **c** CBP shRNA (CBP KD). Blue and red dots represent differential ChIP-seq regions at which read density increased or decreased by 2-fold or more (FDR < 0.05), respectively, in three independent biological replicates. **e**, **f** Fold-change ratio (log_2_) of H3K27ac enrichment at promoters and enhancers in mESCs expressing scramble control (Ctl) shRNA compared with **e** p300 shRNA (p300 KD) or **f** CBP shRNA (CBP KD). Data to the right of log2(0) = 1 indicate regions with increased H3K27ac after acetyltransferase knockdown, while data to the left indicate regions with reduced H3K27ac. *** *p*-value < 2.2e-16 enhancer vs promoter regions

Although lysines in both histones and non-histone proteins are known to be redundantly modified by both CBP/p300 and other acetyltransferases, H3K27 appears to be exclusively acetylated by CBP/p300. To understand the individual contributions of p300 and CBP, which are both expressed in mESCs (Suppl. Figure 1A), to global levels of H3K27ac, we used shRNA to deplete either p300 (p300KD) or CBP (CBPKD) from mESCs. Treatment with multiple shRNA targeting either p300 or CBP resulted in reduction of both the targeted transcript and its protein without greatly affecting the other (Fig. 1a, Suppl. Figure 1B-D) and with little effect on the self-renewal capacity of mESCs (Suppl. Figure 1E, F).

Interestingly, we observed a greater reduction of global H3K27ac levels after depletion of p300 compared to CBPKD (Fig. 1a, Suppl. Figure 1C, D), indicating that p300 is mainly responsible for H3K27ac in mESCs. This result extended to other CBP/p300 target lysines, for example H3K18ac, but not globally to sites targeted by other acetyltransferases, for example H3K9ac or H4K16ac (Suppl. Figure 1G, H). In agreement with published literature (Pasini et al. 2010; Tie et al. 2009), we observed a concomitant increase in global H3K27me3 along with decreased H2K27ac in p300KD ESCs (Fig. 1a). To determine the effect of CBP/p300 loss on H3K27ac genome-wide, we performed chromatin immunoprecipitation followed by sequencing (ChIP-seq) using an H3K27ac antibody. First, we used triplicate analysis to identify all regions of H3K27ac enrichment in wild-type mESCs treated with control shRNAs (*n* = 24,649). We then compared H3K27ac enrichment at these regions in mESCs depleted of either p300 or CBP (Fig. 1b-d), using a 2-fold change cut-off and statistical significance (*p* < 0.05) to identify differentially acetylated regions. In agreement with our global assessment, we found many regions of altered H3K27ac enrichment in p300KD mESCs (Fig. 1b, c, Suppl. Figure 1J), whereas H3K27ac enrichment in CBPKD mESCs was unaffected and strikingly similar to control mESCs (Fig. 1b, d).

As H3K27ac is a mark that correlates with both active enhancers and promoters, we next asked how p300 depletion affected these regions individually. Using H3K27ac enrichment data from control mESCs, we defined regions of promoter enrichment to be within ±3 kb of an annotated transcription start site (*n* = 7336). All other regions were annotated as enhancers (*n* = 16,268). Although both regions showed similar levels of H3K27ac enrichment in control mESCs, only promoters showed high levels of H3K4me3, indicating the validity of this approach (Suppl. Figure 1G). We next compared differences in H3K27ac enrichment at promoters and enhancers in the context of either p300 or CBP depletion (Fig. 1e, f), plotted as a direct comparison of enrichment at specific loci in acetyltransferase-depleted mESCs versus control mESCs (i.e., fold-change values less than log2(0) = 1 represent regions with reduced acetylation in mESCs depleted of p300 or CBP). Interestingly, we found that promoters maintained H3K27ac enrichment to a greater extent (mean log2-fold change at promoters 0.3, at enhancers − 0.24, *p*-value < 2.2e-16) than enhancers when p300 levels are limited (Fig. 1e). Additionally, in agreement with our global and genomic analyses (Fig. 1a-d), we observe little change in either promoter or enhancer H3K27ac enrichment after CBP knockdown compared to control mESCs (mean log2-fold change at promoters 0.13, at enhancers 0.018, p-value < 2.2e-16) (Fig. 1f).

### Chromatin accessibility is independent of CBP/p300 protein levels in mESCs

**Fig. 2:**
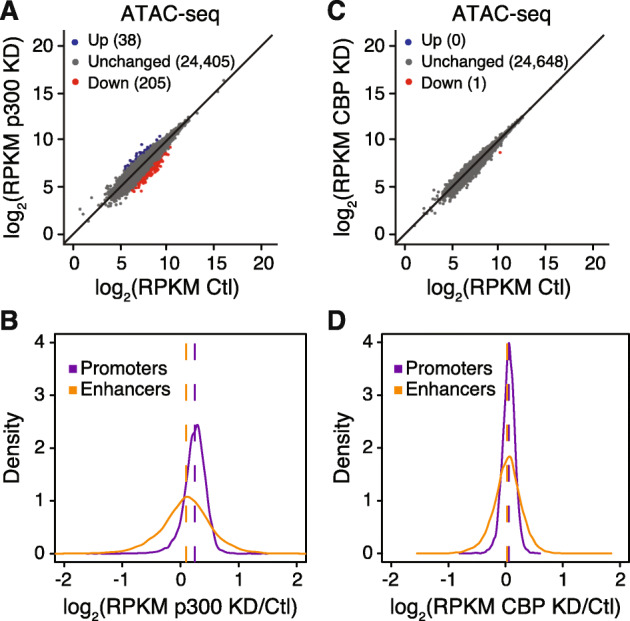
Chromatin Accessibility Is Independent of CBP/p300 Levels in mESCs. **a**, **c** ATAC-seq levels at H3K27ac-enriched regions in mESCs expressing scramble control (Ctl) shRNA compared with **a** p300 shRNA (p300 KD) or **c** CBP shRNA (CBP KD). Blue and red dots represent regions with differential ATAC-seq signal at which read density increased or decreased by 2-fold or more (FDR < 0.05), respectively, in two independent biological replicates. **b**, **d** Fold-change ratio (log_2_) of ATAC-seq enrichment at promoters and enhancers in mESCs expressing scramble control (Ctl) shRNA compared with **b** p300 shRNA (p300 KD) or **d** CBP shRNA (CBP KD). Data to the right of log2(0) = 1 indicate regions with increased ATAC-seq signal after acetyltransferase knockdown, while data to the left indicate regions with reduced ATAC-seq signal

Since we observed reduced H3K27ac at enhancers upon p300 depletion, we next asked whether altering the chromatin modification state would influence the “openness” of these regions. We used ATAC-seq to analyze chromatin accessibility at H3K27ac enriched regions in our control shRNA-treated mESCs as well as mESCs depleted of p300 or CBP (Fig. 2). Differential regions of ATAC-seq enrichment were identified using a 2-fold cut-off and statistical significance (*p* < 0.05). We detected relatively few regions that experienced loss of chromatin accessibility after p300 depletion (Fig. 2a, Suppl. Figure 2A). Moreover, regions with reduced accessibility showed little overlap with regions with reduced H3K27ac (Suppl. Figure 2B). Further analysis comparing either enhancer or promoter ATAC-seq signal in control versus p300KD mESCs confirmed that these regions generally maintained their open state despite the loss of acetylation observed at enhancers (Fig. 2b, Suppl. Figure 2C). In line with CBP having little effect on H3K27ac enrichment, we observed virtually no change in chromatin accessibility upon CBP depletion (Fig. 2c, d). These observations are in agreement with previous reports showing that reduced p300 activity at enhancers under various experimental conditions has little effect on chromatin accessibility in ESCs [19, 21–24]. Our results further demonstrate that high levels of the CBP or p300 protein are not required for maintaining chromatin accessibility.

### Reduced levels of CBP and p300 result in unique transcriptional dysregulation

**Fig. 3:**
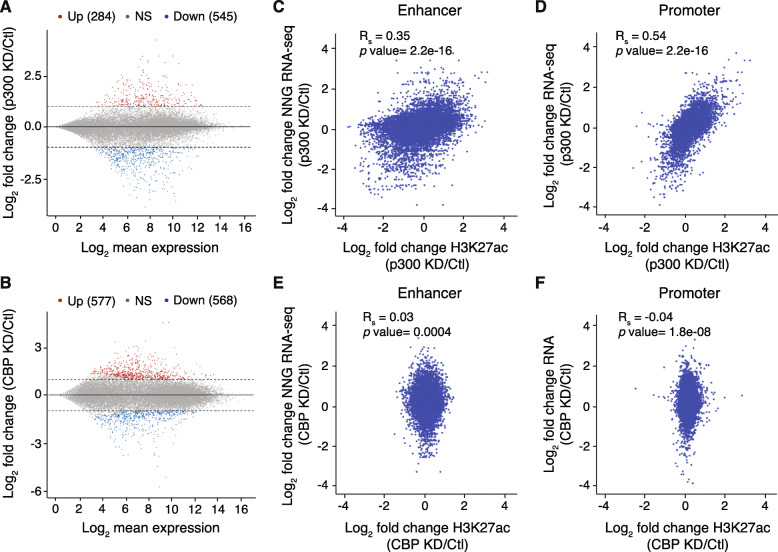
Reduced Levels of CBP and p300 Result in Unique Transcriptional Dysregulation. **a**, **b** MA plot of gene expression in Ctl and **a** p300 KD and **b** CBP KD mESCs. The x-axis indicates gene counts and the y-axis represents the log_2_(fold-change) in expression for KD versus Ctl mESCs. Dotted lines indicate fold-change > 2 and genes in blue and red were differentially expressed (*p* < 0.05). **c**, **e** Correlation plot between enhancer H3K27ac ChIP-seq and nearest-neighboring gene (NNG) RNA-seq for **c** p300 KD and **e** CBP KD represented as log_2_(fold-change) versus Ctl. **d**, **f** Correlation plot between promoter H3K27ac ChIP-seq and RNA-seq of expressed genes (RPKM > 1) for **d** p300 KD and **f** CBP KD represented as log_2_(fold-change) versus Ctl. Spearman correlation tests were performed to determine statistical significance

As CBP and p300 are known transcriptional co-activators, we next wanted to determine how loss of p300 or CBP affects the transcriptional state of the cells. We used RNA-seq to compare transcription levels from equal numbers of control and p300- or CBP-KD mESCs in duplicate using synthetic spike-in standards. We found that the majority of steady-state transcription was unaffected by loss of either p300 or CBP (Fig. 3a, b). Regardless, several hundred genes were dysregulated upon reduction of p300 or CBP in mESCs, resulting in both up- and down-regulation of transcript levels (Fig. 3a, b). Interestingly, we do not observe greater dysregulation of transcription after depletion of p300, the coactivator with a greater effect on H3K27ac.

As transcription and acetylation are strongly correlated, we next compared changes in gene expression to changes in genomic acetylation. We focused our analysis in two ways (Fig. 3c, d). First, we identified the nearest neighboring genes of enhancers identified in our study and compared changes in expression of these genes to changes in acetylation of their putative enhancers. Second, we compared changes in expression for all expressed genes (RPKM > 1) to changes in acetylation at their promoters. p300-dependent changes in acetylation and transcription were moderately correlated when considering promoter acetylation (Rs = 0.54, Fig. 3d), compared to the weak correlation observed between enhancer acetylation and transcription of the nearest neighboring gene (Rs = 0.35, Fig. 3c). Given that very little acetylation in mESCs is dependent upon CBP, it is not surprising that we observed no correlation between enhancer acetylation (Rs = 0.03) or promoter acetylation (Rs = − 0.04) and changes in transcription upon CBP depletion (Fig. 3e, f).

### p53 binding is correlated with maintenance of H3K27ac upon depletion of p300

**Fig. 4:**
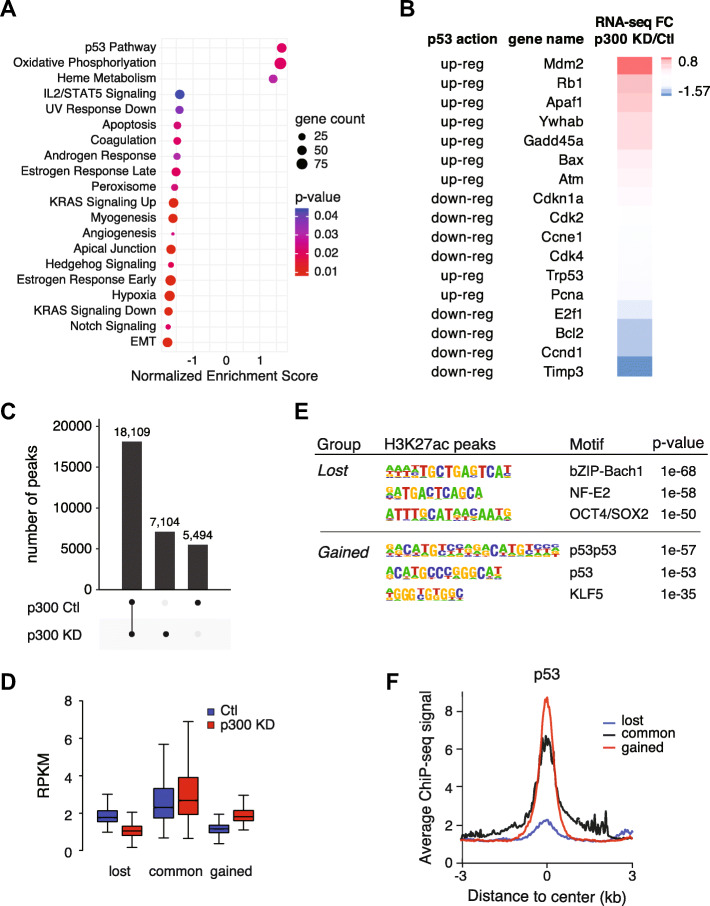
The p53 Pathway Remains Highly Acetylated when p300 Levels are Limited. **a** GSEA pathway analysis of significantly regulated genes (based on RNA-seq) in p300 KD mESC compared to Ctl mESC. The normalized enrichment score (NES) is indicated for each gene set. **b** Fold-change RPKM (RNA-seq) of p300 KD versus Ctl for genes belonging to the p53 pathway. Effect of p53 on gene expression is indicated. **c** UpSet representation of regions of H3K27ac enrichment and overlap in Ctl and p300 KD mESCs. **d** Boxplot showing H3K27ac enrichment at lost, common and gained peaks after p300 KD. *p* < 2.2 × 10^− 16^ for all comparisons by Wilcoxon rank sum test. The bottom and top of the boxes correspond to the 25th and 75th percentiles, and the internal band is the 50th percentile (median). The plot whiskers correspond to 1.5× interquartile range and outliers are excluded. **e** Motif enrichment analysis of lost and gained regions of H3K27ac enrichment after p300 KD. Logo and *p*-values are indicated for each motif. **f** Average profile of p53 ChIP-seq in WT mESCs at lost, common and gained regions of H3K27ac enrichment after p300 KD

As we observed that p300 and CBP regulate different sets of genes in mESCs (Suppl. Figure 3A), we performed gene set enrichment analysis (GSEA) of our RNA-seq data to understand the pathways regulated by p300 and CBP, respectively (Fig. 4a, Suppl. Figure 3B). Surprisingly, one of the most enriched terms observed after p300 knockdown was the p53 pathway (Fig. 4a), further confirmed by dysregulation of several p53 targets (Fig. 4b). This pathway was not observed in GSEA analysis of CBP-regulated genes (Suppl. Figure 3B). Although p53 is a known target of p300 [25–27] we did not observe decreased p53 acetylation upon p300 depletion (Suppl. Figure 3C). Further, while we cannot rule out a general stress response after p300 knockdown, we did not observe clear signs of baseline DNA damage or sensitivity to irradiation after depletion (Suppl. Figure 3D), nor alteration of cell cycle after p300 knockdown in mESC (Suppl. Figure 3E).

To corroborate our transcriptional analysis, we returned our focus to H3K27ac enrichment profiles in control and p300KD mESCs. Using peak calling tools, we identified regions of H3K27ac enrichment that were identified in our control mESCs (and therefore lost after p300 depletion, referred to as lost), regions that were common to both the control and the p300KD mESCs (common), and regions that were enriched in p300KD mESCs (gained) (Fig. 4c). We validated our peak calling by demonstrating the expected changes in H3K27ac enrichment at these regions in control and p300KD mESCs (Fig. 4d). These regions are all enriched with p300 in control ESCs (Suppl. Figure 4A), supporting our conclusion that p300 is responsible for this activity. Further, all regions are enriched with both H3K4me1 and H3.3, indicative of active regulatory elements (Suppl. Figure 4B). Importantly, we find that regions with reduced H3K27ac in p300KD ESCs are lowly enriched with H3K4me3 compared to regions that maintain or gain H3K27ac (Suppl. Figure 4B), in alignment with our assessment that enhancers are more likely to lose H3K27ac when p300 levels are limited than promoters.

To determine regulatory networks underlying H3K27ac enrichment in control and p300KD mESCs, we used HOMER [28] to predict transcription factor binding motifs enriched at these regions. In regions at which H3K27ac is reduced upon p300 depletion, one of the most enriched motifs was Oct4/Sox2 (*p* = 1 × 10^− 50^), which is highly correlated with pluripotency (Fig. 4e). Likewise, these regions are highly enriched with the pluripotency transcription factors, Oct4, Sox2, and Nanog [29], in wild-type mESCs (Suppl. Figure 4C). Interestingly, we found that regions with increased H3K27ac (gained) upon p300 depletion were enriched with the p53 binding motif (Fig. 4e). Finally, we performed p53 ChIP-seq in wild-type mESCs to determine p53 enrichment across these regions. We found that p53 was greatly enriched in the regions that gain acetylation upon p300 depletion compared to regions that lose H3K27ac when p300 levels are reduced (Fig. 4f). Further, pluripotency transcription factors showed relatively low enrichment at regions that gain H3K27ac after p300 depletion (Suppl. Figure 4C). Interestingly, and in line with previous studies suggesting pioneer factor activity for p53 under specific contexts [30, 31], we observe that the “gained” p53-bound regions exist in a more closed state compared with the regions highly bound by pluripotency transcription factors in control mESCs (Suppl. Figure 4D).

## Discussion

We provide here, to our knowledge, the first differential genomic analysis of CBP/p300 enzymatic activity on chromatin in mESCs, using H3K27ac ChIP-seq, ATAC-seq, and RNA-seq to determine the unique effects of CBP or p300 depletion on chromatin state and associated gene expression in ESCs. We find that mESCs are quite tolerant of reduced H3K27ac at enhancers, and that promoter acetylation is a stronger indicator of transcriptional competence. Further, we find that p53-bound regulatory elements largely maintain H3K27ac when p300 levels are reduced.

Interestingly, we find that the regions that lose the most H3K27ac when p300 levels are limited are also regions that are highly bound by pluripotency-specific transcription factors. This observation is in line with the idea that genes with greater local concentration and diversity of coactivators are less reliant on CBP/p300 to maintain steady state gene expression [32]. Although p300 and CBP function is described at both promoters and enhancers [2] our findings indicate that promoters experience less disruption of H3K27ac when p300 levels are reduced. This observation is in agreement with patterns of H3K27ac loss after treatment with CBP/p300 bromodomain inhibitor [23]. Previous studies have interpreted results such as these as an indication that CBP/p300 play a more important role at enhancers. We suggest that when p300 levels are limited, loss of enhancer acetylation is tolerated while maintenance of promoter acetylation is more important to safeguard critical cell functions. In support, we observe stronger correlation between changes in promoter acetylation and transcription than changes in enhancer acetylation and transcription of the nearest neighboring genes upon depletion of p300, although it is possible that this weak correlation is due to the imperfect nature of assigning an enhancer to its target gene in this way [33]. It is also formally possible and intriguing to consider that an enzyme other than CBP/p300 is responsible for H3K27ac at promoters.

Given the high correlation between the “open” chromatin state and histone acetylation, we were surprised that reduced p300 and the subsequent reduction in H3K27 acetylation did not affect chromatin accessibility at acetylated regions. Nevertheless, this data aligns with our previous observation that a significant reduction of p300 HAT activity (i.e., H3K27ac) fails to “close” chromatin at p300 targets [19]. It is also in agreement with a study in which small-molecule inhibition of the CBP/p300 bromodomain reduced global H3K27ac without changing the open chromatin state [23] and further supported by study replacing endogenous H3.3K27 with H3.3K27R in ESCs, resulting in dramatic loss of H3K27ac at enhancers with virtually no change in chromatin accessibility [21]. Taken together, these data suggest that neither the HAT activity of p300 nor more fundamentally its presence is required to maintain open chromatin at regulatory regions. Interestingly, we find that the regions that lose the most H3K27ac when p300 levels are limited are also regions that are highly bound by pluripotency-specific transcription factors. This observation is in line with previous data suggesting that genes with greater local concentration and diversity of coactivators are less reliant on CBP/p300 to maintain steady state gene expression [18]. Further, it is possible that loss of H3K27ac alone is not sufficient to reduce chromatin accessibility given the myriad other acetylations (both histone and transcription factor) present at enhancers.

Perhaps surprising for knockdown of a transcriptional co-activator, we observe just over 1000 regions with increased H3K27ac as well as hundreds of up-regulated transcripts upon p300 depletion. One explanation could be that residual p300 after knockdown can be redirected to select regions and favor both acetylation and transcription of specific targets. Intriguingly, regions that maintain or gain acetylation when p300 is limited are enriched with p53 binding motifs and are indeed highly bound by p53 in wild-type mESCs. In agreement with previous studies [30, 31], we find that p53-bound enhancers are located within regions of relatively less accessible chromatin, suggesting that p53 may regulate enhancer activity, in part, by modulating chromatin accessibility given the appropriate cellular context.

Taken together, our study offers new insights into an important family of transcriptional coactivators. What still remains is the question of why CBP and p300 have seemingly different activity in mESCs. As a previous study demonstrates very similar patterns of enrichment between p300 and CBP in mESCs [34], it is unlikely that our results can be explained by differential enrichment profiles. In order to address this question, future studies will determine both the interactome of CBP and p300 in mESCs as well as the broader protein acetylome that can be attributed to each enzyme.

## Conclusions

We find that p300, and not CBP, is responsible for maintaining H3K27ac at regulatory elements in mESCs. p300-mediated acetylation of promoters correlates more strongly with transcription than enhancer acetylation, and changes in acetylation do not correlate with changes in DNA accessibility. Finally, we find that regions that maintain H3K27ac after p300 depletion are enriched with p53 binding motifs. Taken together, our study offers new insights into an important family of transcriptional coactivators that play key roles in both development and human disease.

## Methods

### mESC culture

Mouse embryonic stem cell lines (mESCs) (C57Bl/6 J background) (gift from D. Wen at Weill Cornell Medical College) were cultured on gelatin-coated plates under standard serum/LIF conditions at 37 °C with 5% CO_2_ (KO-DMEM, 2 mM Glutamax, 15% ES grade fetal bovine serum, 0.1 mM 2-mercaptoethanol, 1x Pen/Strep, 1x NEAA and leukemia inhibitory factor (LIF)). During thawing and early passages, cells were maintained on an irradiated feeder layer. To remove feeders, cells were passaged at least two passages off of feeders onto gelatin-coated plates. mESCs were routinely tested for mycoplasma.

### shRNA transduction

For p300 and CBP knockdown, 5 μg of plasmids (Dharmacon) were packaged with 5 μg psPAX2, and 0.5 μg VSVG plasmids and transfected in serum-free media into 3 × 10^6^ 293 T cells in a 10 cm^2^ tissue culture dish using Lipofectamine 3000. Lentivirus-containing supernatants were harvested 48 and 72 h post-transfection, pooled, and concentrated 10x with Lenti-X (Clontech, 63,123). 2 × 10^5^ WT mESCs were incubated with 0.2 ml concentrated lentivirus and polybrene (8 μg/ml). The next day, the media was replaced with complete mESC culture media containing 1 μg/ml puromycin (for p300 plasmid) or 400 μg/ul (for CBP plasmid) of G418. After 4 days of selection, mESCs were used for downstream analysis.

### Antibodies

H3K27ac (39,133, Active Motif Lot # 31814008), H3K27ac (39,685, Active Motif, no. 14517014), H3K18ac (ab1191, Abcam, no. GR3211480–1), H3K27me3 (9756, Cell Signaling), H3K9ac (ab4441, Abcam), H4K16ac (39,167, Active Motif), H3 general (ab1791, Abcam, no. GR177884–2), Spike-In antibody (61,686, Active Motif, Lot# 00419007), p300 (sc-584, Santa Cruz, Lot # F3016), Gapdh (2118, Cell Signaling, Lot # 10), CBP(D6C5) (7389S, Cell Signaling), p53(CM5) (NCL-L-p53-CM5p, Leica Biosystems), PKCs p2056 (ab18192, Abcam), KAP1/TRIM29 pS824 (A300-767A, Bethyl), Acetyl-p53 (Lys379) (2570, Cell Signaling), anti-mouse IgG-HRP (NA93V, GE, no.9773218), anti-rabbit IgG-HRP (170–6515, Bio-Rad, no. 350003248).

### Irradiation of cells

Gamma radiation experiments were conducted at UT Southwestern Medical Center using a 137Cs source irradiator. mESC cells were irradiated with 10 Gy of irradiation (IR) for 3 min and allowed to recover for 30 min.

### Cell cycle analysis

2 × 10^^6^ cells for each cell line were incubated with BrdU at a final concentration of 10 μM for 20 min and then processed following manufacturer’s instructions (BD Pharmingen™ BrdU Flow Kits).

### Chromatin Immunoprecipitation (ChIP)

Native (for histone PTMs) and crosslinking (X) (for p300) ChIP were performed with 5 × 10^6^ (in triplicate) and 1 × 10^8^ cells (one replicate), respectively, as previously described [19] with slight modifications. A spike-in normalization strategy was used to normalize all ChIP-seq data to reduce the effects of technical variation and sample processing bias. Spike-In chromatin (Active Motif, 53,083) and Spike-in antibody (Active Motif, 61,686) were used according to manufacturer instructions.

#### Native ChIP

Cells were trypsinized, washed and subjected to hypotonic lysis (50 mM TrisHCl pH 7.4, 1 mM CaCl_2_, 0.2% Triton X-100, 10 mM NaButyrate, and protease inhibitor cocktail (Roche)) with micrococcal nuclease for 5 min at 37 °C to recover mono- to tri-nucleosomes. Nuclei were lysed by brief sonication and dialyzed into RIPA buffer (10 mM Tris pH 7.6, 1 mM EDTA, 0.1% SDS, 0.1% Na-Deoxycholate, 1% Triton X-100) for 2 h at 4 °C. Soluble material was incubated with 3–5 μg of antibody bound to 50 μl protein A or protein G Dynabeads (Invitrogen) and incubated overnight at 4 °C, with 5% reserved as input DNA. Magnetic beads were washed as follows: 3x RIPA buffer, 2x RIPA buffer + 300 mM NaCl, 2x LiCl buffer (250 mM LiCl, 0.5% NP-40, 0.5% NaDeoxycholate), 1x TE + 50 mM NaCl. Chromatin was eluted and treated with RNaseA and Proteinase K. ChIP DNA was purified and dissolved in H_2_O.

#### X-ChIP

Cells were crosslinked with 1% formaldehyde for 10 min at room temperature and quenched with 0.125 M glycine. Cells were divided in 3 batches and ChIPs were performed in parallel and then pooled together. Cells were resuspended in Farnham Lysis Buffer (5 mM PIPES pH 8, 85 mM KCl, 0.5% NP-40, 1 mM DTT, protease and phosphatase inhibitors) and isolated nuclei in Lysis Buffer (1% SDS, 10 mM EDTA, 50 mM Tris (pH 7.9), 1 mM DTT, protease and phosphatase inhibitors) and chromatin was sonicated to an average size of 0.3–0.7 kb using Covaris M220 Focused-ultrasonicator. Soluble material was diluted in 10X dilution buffer (0.5% Triton X-100, 2 mM EDTA, 20 mM Tris (pH 7.9), 150 mM NaCl, 1 mM DTT, protease and phosphatase inhibitors) and incubated with 40 μl of antibody bound to 150 μl protein A/G mixed Dynabeads (Invitrogen) and incubated overnight at 4 °C, with 5% reserved as input DNA. Magnetic beads were washed as follows: 1x Low Salt buffer (10 mM TrisHCl pH 8, 2 mM EDTA, 0.1% SDS, 1% Triton X-100, 150 mM NaCl), 1x High Salt buffer (10 mM TrisHCl pH 8, 2 mM EDTA, 0.1% SDS, 1% Triton X-100, 500 mM NaCl), 1x LiCl buffer (10 mM TrisHCl pH 8, 1 mM EDTA, 1% NP-40, 1% NaDeoxycholate, 250 mM LiCl), 1x TE + 50 mM NaCl. Chromatin was eluted and treated with RNaseA and Proteinase K. ChIP DNA was purified and dissolved in H_2_O.

#### ChIP–qPCR

qPCR was performed in triplicate using a LightCycler 480 Instrument II system and Power SYBR Green PCR master mix. ChIP DNA samples were diluted 1:10 in H2O, with 5 μl used per reaction. ChIP–qPCR signal is represented as percent input in Supplemental Figure 1J and plotted as direct comparison with Ctl. All qPCR primer sequences used in this study are listed in Supplementary Table 3.

### ChIP-seq

#### ChIP-seq library preparation

ChIP-seq libraries were prepared from 5 ng ChIP DNA following the Illumina TruSeq protocol. The quality of the libraries was assessed using a D1000 ScreenTape on a 2200 TapeStation (Agilent) and quantified using a Qubit dsDNA HS Assay Kit (Thermo Fisher). Libraries with unique adaptor barcodes were multiplexed and sequenced on an Illumina NextSeq 500 (paired-end, 33 base pair reads). Typical sequencing depth was at least 30 million reads per sample.

#### ChIP-seq data quality control, alignment and spike-in normalization

Quality of ChIP-seq datasets was assessed using the FastQC tool. ChIP-seq raw reads were aligned separately to the mouse reference genome (mm10) and the spike-in drosophila reference genome (dm3) using BWA [35]. Only one alignment is reported for each read (either the single best alignment or, if more than one equivalent best alignment was found, one of those matches selected randomly). Duplicate reads were filtered using the Picard MarkDuplicates. Uniquely mapped drosophila reads were counted in the sample containing the least number of drosophila mapped reads and used to generate a normalization factor for random downsampling. Reads were converted into bigWig files using BEDTools (v2.29.0) [36] for visualization in Integrative Genomics Viewer.

#### Downstream ChIP-seq analysis

- *Peak Calling*. Peak calling was performed using MACS14 [37] software using input as a control in each replicated sample. HOMER mergePeaks [28] was used to get unique peaks from replicates to reduce false positives and retain only robust peaks for further analyses.
- *Average Profiles.* Bigwig files were used to generate average ChIP-seq profiles using deepTools.
- *Heatmaps.* The read densities surrounding 6 kb (± 3 kb) of the peak center of WT H3K27ac peaks were determined and visualized as heatmaps using deepTools [38].
- *Box plots.* Box plot representations were used to quantitatively assess the read distribution in a fixed window. Box plots are defined by the median, box limits at upper and lower quartiles of 75 and 25%, and whiskers at 90 and 10%. The read distribution surrounding the peak center was calculated and plotted using custom R scripts. Wilcoxon rank sum tests were performed to determine the statistical significance of all comparisons.
- *Differential Expression Analysis (DE) of H3K27ac ChIP-seq.* Differential peaks were found using multiBamSummary (DeepTools) [38] in a BED-file mode, by using WT H3K27ac peaks as input BED file. The output of multiBamSummary is a compressed numpy array (.npz) that was directly used by the program DE-Seq2 with fold change ≥2 or ≤ − 2, FDR < 0.05 to calculate and visualize pairwise correlation values between the read coverages using custom R scripts.
- *Density plots.* Density plots representing fold-change differences between samples were generated using custom R scripts. k-s tests were performed to determine the statistical significance of all comparisons.
- *UpSet Plots*. To check the extent of overlap of H3K27ac peaks between samples, UpSet Plots were generated using custom R scripts.
- *Motif analysis.* HOMER findMotifs [28] was used to perform motif analysis on H3K27ac peaks.

### ATAC-seq

ATAC-seq was performed in duplicate as previously described [39] with minor changes. For each sample, 100,000 cells were harvested, washed and lysed with ATAC buffer (Tris 10 mM pH 7.4, 10 mM NaCl, 3 mM MgCl_2_, NP-40 0.1%, Tween-20 0.1%, Digitonin 0.01%). Nuclei were collected and subject to tagmentation at 37 °C for 30 min in adjusted tagmentation buffer (2x TD Tagment buffer + Digitonin 0.01% + 5 ul of TDE Tagment DNA enzyme from Illumina). Reaction was stopped with 0.2% SDS and DNA was collected using Qiaquick PCR purification columns and eluted in 10 μl 10 mM Tris, pH 8. Eluted DNA was amplified using NebNext Q5 MM kit and purified using AMPure XP beads (negative and positive selection). Samples were pooled for multiplexing and sequenced using paired-end sequencing on the Illumina NextSeq 500.

#### ATAC-seq data quality control, alignment and normalization

Quality of the ATAC-seq datasets was assessed using the FastQC tool. The ATAC-seq reads were then aligned to the mouse reference genome (mm10) using BWA [35]. For unique alignments, duplicate reads were filtered out. The resulting uniquely mapped reads were normalized to the same read depth across all samples and converted into bigWig files using BEDTools [36] for visualization in Integrative Genomics Viewer [40]. Heatmaps were generated using deepTools.

#### Downstream ATAC-seq analysis

- *Differential Expression Analysis (DE) of ATAC-seq.* Differential peaks were found using multiBamSummary (DeepTools) [38] in a BED-file mode, by using WT H3K27ac peaks as BED file. The output of multiBamSummary is a compressed numpy array (.npz) that was directly used by the program DE-Seq2 with fold change ≥2 or ≤ − 2, FDR < 0.05 to calculate and visualize pairwise correlation values between the read coverages using custom R scripts.
- *Density plots.* Density plots representing fold-change differences between samples were generated using custom R scripts. k-s tests were performed to determine the statistical significance of all comparisons.

### Quantitative RT-PCR and mRNA-seq

mRNA was isolated using QIAGEN RNeasy. 500 ng of total RNA was reverse transcribed using random hexamers and MultiScribe reverse transcriptase. mRNA expression was analyzed by quantitative PCR (qPCR) with SYBR Green using a LightCycler 480 (Roche). All qPCR primer sequences used in this study are listed in Table S3.

For RNA-seq, total mRNA from two biological replicates and from equal cell numbers was mixed with synthetic RNA standards (ERCC RNA Spike-In Mix, Thermo Fisher) [41]. Libraries were prepared according to the Illumina TruSeq protocol and sequenced on an Illumina NextSeq 500 (paired-end, 33 base pair reads).

### Analysis of RNA-seq data

#### Data quality control, alignment and normalization

Quality of the RNA-seq raw reads was assessed using the FastQC tool. The reads were then aligned to the mouse reference genome (mm10) and the spike-in control ERCC92 using STAR [42]. Reads mapping to ERCC92 were counted using htseq-count [43] and used to normalize the counts to genes. After normalization, the reads were converted into bigWig files using BEDTools for visualization in Integrative Genomics Viewer [40] or the UCSC genome browser.

#### Downstream RNA-seq analysis

- *Differential Expression Analysis (DE)*. Gene expression level measured as FPKM was determined by the maximum likelihood estimation method implemented in the htseq-count software package with annotated transcripts as references. Differential expression was analyzed using the Student’s t test in the program DE-Seq2 with *p* values corrected for multiple testing.
- *MA Plots.* MA plots were used to graphically represent genes that were upregulated or downregulated by more than 2-fold. The log2 fold change (KO/WT) was plotted on the y-axis versus the log2 mean of normalized counts on the x-axis.
- *Venn Diagrams*. To check the extent of overlap of the Up and Down regulated genes from RNA-seq from p300 and CBP datasets, venn diagrams were generated using custom R scripts.
- *Correlation plots.* Correlation of fold-change differences between samples, comparing H3K27ac levels and RNA expression was generated using custom R scripts. Spearman correlation tests were performed to determine the statistical significance of all comparisons.

### Quantification and statistical analysis

To check the significance of all comparisons, Wilcoxon rank sum test was used to calculate *p*-values for data used to generate boxplots. Two-sample Kolmogorov-Smirnov test was used to calculate p-values to show significant changes between two density curves. Spearman correlation tests were performed to determine the statistical significance of correlation plots. T-student test was used for the western blot quantification, where the data were expressed as mean ± S.D. of at least 3 independent experiments.

### Code availability

Codes to generate figures are available upon request.

### Data sets

The following published next-generation sequencing data sets were meta-analysed in this study: 1) ChIP-seq of H3K4me1, H3K4me3, p300, H3.3 in WT ESCs [19]. 2) ChIP-seq of Oct4, Nanog, Sox2 in WT ESCs [29]. 3) ChIP- Seq of CBP [20].

## Supplementary information

**Additional file 1: Figure S1.** p300 Maintains Enhancer Acetylation in mESCs. (A) Log_2_(FPKM) expression of transcripts in WT mESC cells quantified by RNA-seq of two biological replicates, showing the expression of p300 and CBP. Horizontal dashed line represents the median expression level. (B) Transcript levels (RT-qPCR) of p300 and CBP in mESCs transfected with scramble shRNA or either p300 or CBP shRNA. *** *p* < 0.001 vs Ctl.(C) Immunoblot of whole cell lysates from mESCs transfected with scramble shRNA of either p300 or CBP shRNA. Blot is representative of three independent experiments. (D) Quantification of western blot in panel C represented as mean (*n* = 3) ± s.d. *** p < 0.001 vs Ctl. (E) Transcript levels (RT-qPCR) of Oct4 and Nanog in p300 and CBP KD mESCs compared to Ctl. (F) Alkaline phosphatase staining of Ctl, p300 KD, and CBP KD mESCs in S/L media. Scale bar = 1 mm. (G) Immunoblot of whole cell lysates from mESCs transfected with scramble shRNA or either p300 or CBP shRNA. Blot is representative of three independent experiments. (H) Quantification of western blot in panel G represented as mean (n = 3) ± s.d. (I) Boxplot showing H3K27ac (left), H3K4me3 [19] (center) and H3K4me1 [19] (right) enrichment at enhancers (*n* = 16,268) and promoters (*n* = 7336) in wild-type cells. *p* < 2.2 × 10^− 16^ for all comparisons by Wilcoxon rank sum test. The bottom and top of the boxes correspond to the 25th and 75th percentiles, and the internal band is the 50th percentile (median). The plot whiskers correspond to 1.5× interquartile range and outliers are excluded. (J) H3K27ac ChIP-qPCR validation from two different p300KD hairpins of H3K27ac enrichment at gained (left) and lost (right) regions compared to the corresponding control mESCs.

**Additional file 2: Figure S2.** Chromatin Accessibility Is Independent of p300/CBP Levels in mESCs. (A) Heatmap of ATAC-seq in Ctl and p300 KD cells at regions that lose (down), maintain (no change), and gain (up) H3K27ac after p300 KD. Each row represents a single region. (B) Venn diagrams showing the relationship between ATAC-seq and ChIP-seq dysregulated regions after p300 KD. (C) ChIP-seq heatmap of H3K27ac at enhancers (left) and promoters (right) in Ctl and p300 KD mESCs.

**Additional file 3: Figure S3.** H3K27ac is maintained at p53 motifs after p300 depletion. (A) Venn diagrams showing the relationship between p300 KD and CBP KD RNA-seq in up- (top) and down-regulated (bottom) genes. (B) GSEA pathway analysis of significantly regulated genes (based on RNA-seq) in CBP KD mESC compared to Ctl mESC. The normalized enrichment score (NES) is indicated for each gene set. (C) Immunoblot from whole cell lysates from mESCs transfected with scramble shRNA (Ctl) or p300 shRNA. Blot is representative of three independent experiments. (D) Immunoblot from whole cell lysates from mESCs transfected with scramble shRNA (Ctl) or p300 shRNA. Prior to harvesting, mESCs were treated for three min with 10 Gy of IR. Blot is representative of three independent experiments. (E) Cell cycle phase distribution for Ctrl and KD cells. Cells were stained with BrdU and 7-AAD according to the kit manufacturer’s instructions (BD Pharmingen™ BrdU Flow Kits). Graph shows quantification and standard deviation over three independent experiments.

**Additional file 4: Figure S4.** Chromatin features of p300-dependent enhancers in wild-type mESCs. (A) ChIP-seq average profiles of p300 [19] in wild-type mESCs at regions of H3K27ac enrichment that are lost, common and gained after p300 KD. (B) ChIP-seq average profiles of H3K4me1, H3K4me3 and H3.3 [19] in wild-type mESCs at regions of H3K27ac enrichment that are lost, common and gained after p300 KD. (C) ChIP-seq average profiles of Oct4, Nanog, Sox2 [29] in wild-type mESCs at regions of H3K27ac enrichment that are lost, common and gained after p300 KD. (D) ATAC-seq average profile in wild-type mESCs at regions of H3K27ac enrichment that are lost, common and gained after p300 KD.

**Additional file 5: Figure S5.** Raw immunoblots. (A) Western blots related to Fig. 1a. (B) Western blots related to Supplementary Figure 1C and 1G. (C) Western blots related to Supplementary Figure 3C and 3D.

**Additional file 6.**

**Additional file 7.**

**Additional file 8.**

**Additional file 9.**

**Additional file 10.**

## Data Availability

Datasets are deposited in the NCBI Gene Expression Omnibus using the Series reference: GSE138925. Requests for materials should be directed to laura.banaszynski@utsouthwestern.edu.

## Abbreviations

CBP: CREB binding protein
mESC: Mouse Embryonic Stem Cells
ChIP-seq: Chromatin Immunoprecipitation followed by sequencing
ATAC-seq: Assay for Transposase-Accessible Chromatin followed by sequencing
GSEA: Gene Set Enrichment Analysis
