# Supplementary figures and images for: Differential contribution of p300 and CBP to regulatory element acetylation in mESCs
Source: BMC Mol Cell Biol. 2020 Jul 20;21:55. doi: 10.1186/s12860-020-00296-9 (PMC7370441; doi:10.1186/s12860-020-00296-9)

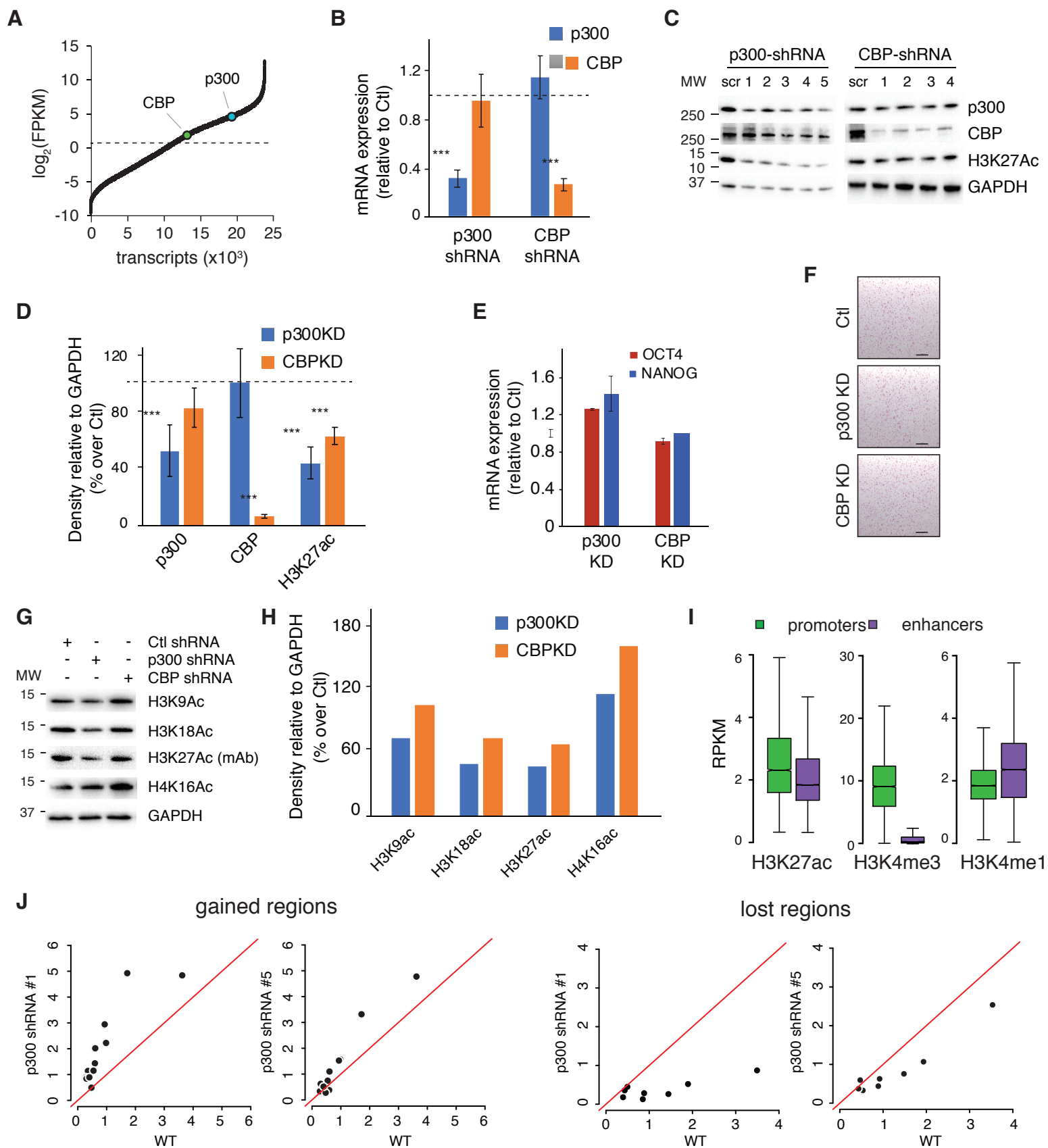

Supplement: Supplementary file 1 — Additional file 1: Figure S1. p300 Maintains Enhancer Acetylation in mESCs. (A) Log2(FPKM) expression of transcripts in WT mESC cells quantified by RNA-seq of two biological replicates, showing the expression of p300 and CBP. Horizontal dashed line represents the median expression level. (B) Transcript levels (RT-qPCR) of p300 and CBP in mESCs transfected with scramble shRNA or either p300 or CBP shRNA. *** p < 0.001 vs Ctl.(C) Immunoblot of whole cell lysates from mESCs transfected with scramble shRNA of either p300 or CBP shRNA. Blot is representative of three independent experiments. (D) Quantification of western blot in panel C represented as mean (n = 3) ± s.d. *** p < 0.001 vs Ctl. (E) Transcript levels (RT-qPCR) of Oct4 and Nanog in p300 and CBP KD mESCs compared to Ctl. (F) Alkaline phosphatase staining of Ctl, p300 KD, and CBP KD mESCs in S/L media. Scale bar = 1 mm. (G) Immunoblot of whole cell lysates from mESCs transfected with scramble shRNA or either p300 or CBP shRNA. Blot is representative of three independent experiments. (H) Quantification of western blot in panel G represented as mean (n = 3) ± s.d. (I) Boxplot showing H3K27ac (left), H3K4me3 [19] (center) and H3K4me1 [19] (right) enrichment at enhancers (n = 16,268) and promoters (n = 7336) in wild-type cells. p < 2.2 × 10− 16 for all comparisons by Wilcoxon rank sum test. The bottom and top of the boxes correspond to the 25th and 75th percentiles, and the internal band is the 50th percentile (median). The plot whiskers correspond to 1.5× interquartile range and outliers are excluded. (J) H3K27ac ChIP-qPCR validation from two different p300KD hairpins of H3K27ac enrichment at gained (left) and lost (right) regions compared to the corresponding control mESCs. [file 12860_2020_296_MOESM1_ESM.pdf]

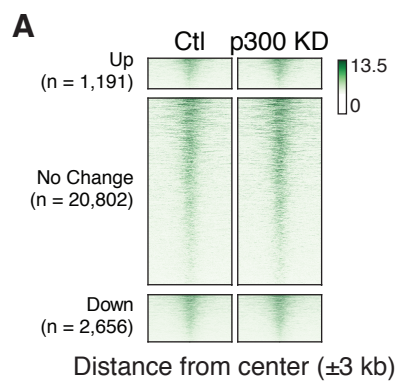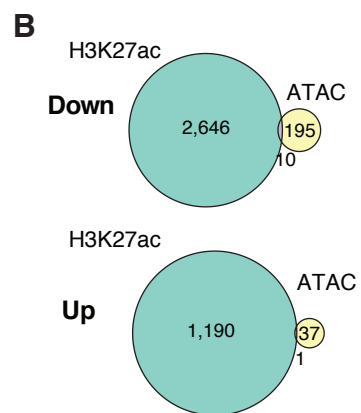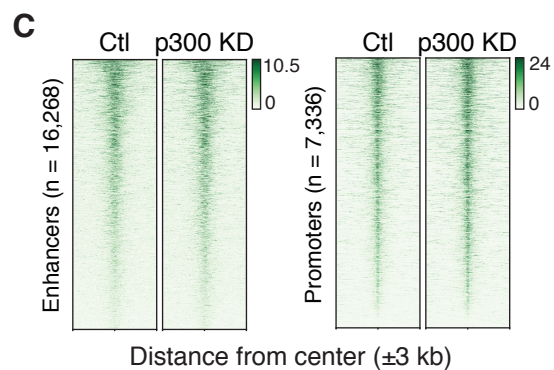

Supplement: Supplementary file 2 — Additional file 2: Figure S2. Chromatin Accessibility Is Independent of p300/CBP Levels in mESCs. (A) Heatmap of ATAC-seq in Ctl and p300 KD cells at regions that lose (down), maintain (no change), and gain (up) H3K27ac after p300 KD. Each row represents a single region. (B) Venn diagrams showing the relationship between ATAC-seq and ChIP-seq dysregulated regions after p300 KD. (C) ChIP-seq heatmap of H3K27ac at enhancers (left) and promoters (right) in Ctl and p300 KD mESCs. [file 12860_2020_296_MOESM2_ESM.pdf]

**A**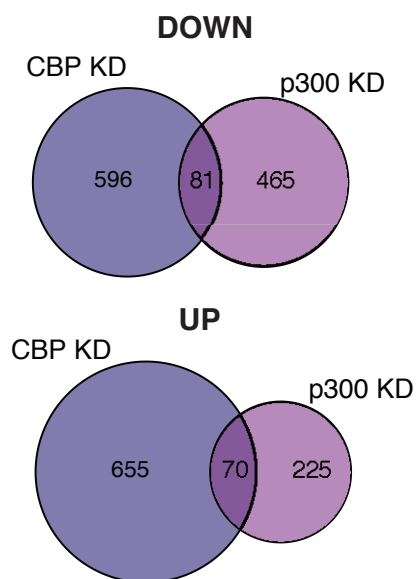**B**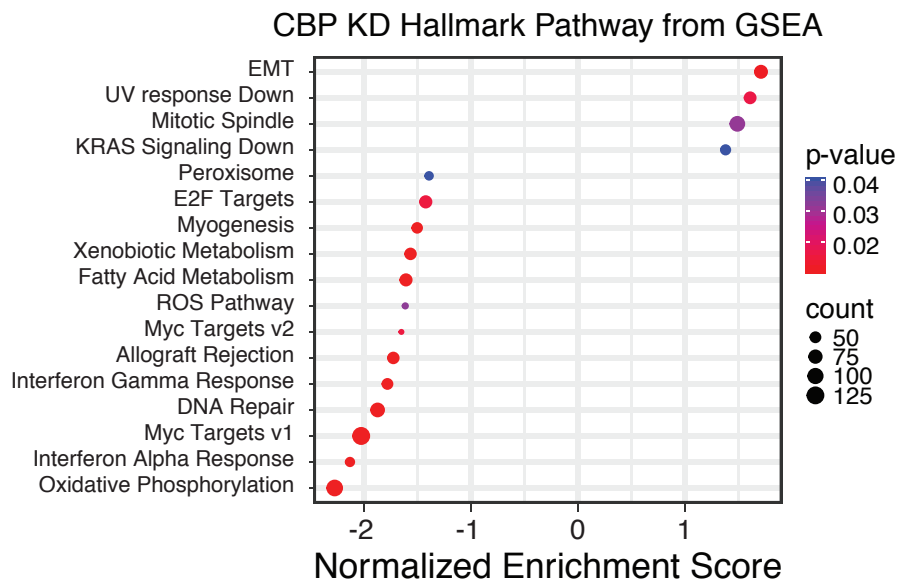**C**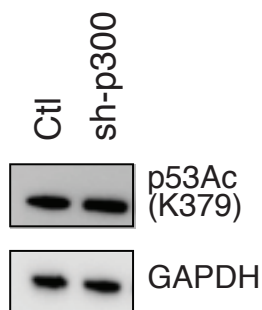**D**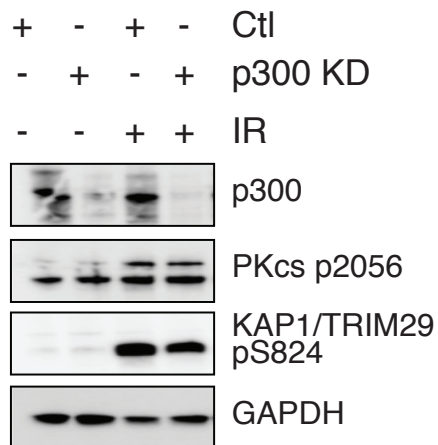**E**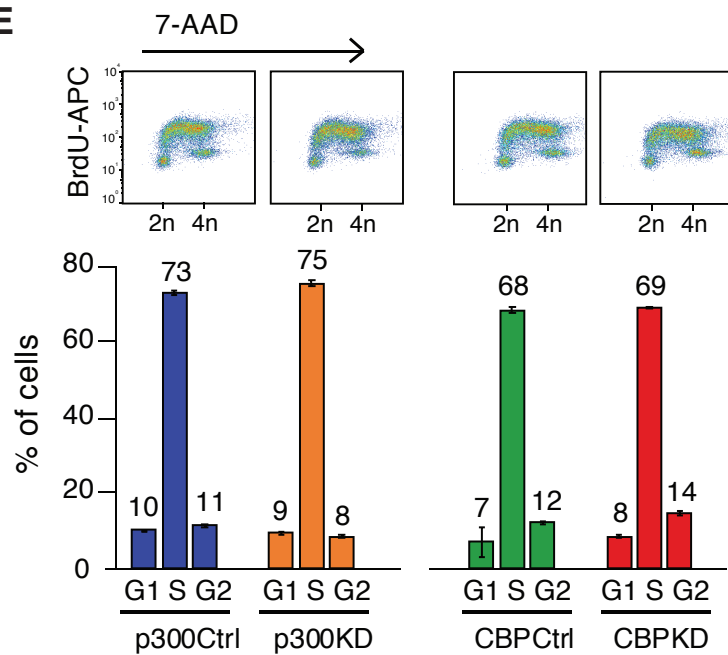

Supplement: Supplementary file 3 — Additional file 3: Figure S3. H3K27ac is maintained at p53 motifs after p300 depletion. (A) Venn diagrams showing the relationship between p300 KD and CBP KD RNA-seq in up- (top) and down-regulated (bottom) genes. (B) GSEA pathway analysis of significantly regulated genes (based on RNA-seq) in CBP KD mESC compared to Ctl mESC. The normalized enrichment score (NES) is indicated for each gene set. (C) Immunoblot from whole cell lysates from mESCs transfected with scramble shRNA (Ctl) or p300 shRNA. Blot is representative of three independent experiments. (D) Immunoblot from whole cell lysates from mESCs transfected with scramble shRNA (Ctl) or p300 shRNA. Prior to harvesting, mESCs were treated for three min with 10 Gy of IR. Blot is representative of three independent experiments. (E) Cell cycle phase distribution for Ctrl and KD cells. Cells were stained with BrdU and 7-AAD according to the kit manufacturer’s instructions (BD Pharmingen™ BrdU Flow Kits). Graph shows quantification and standard deviation over three independent experiments. [file 12860_2020_296_MOESM3_ESM.pdf]

**A**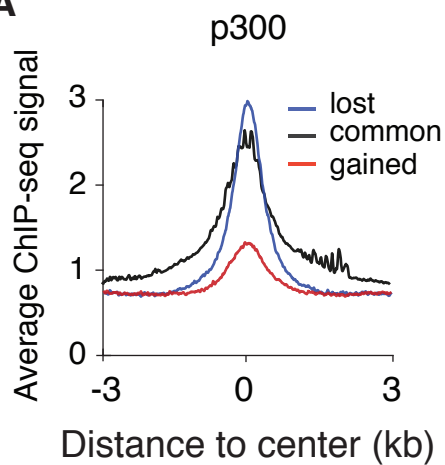**B**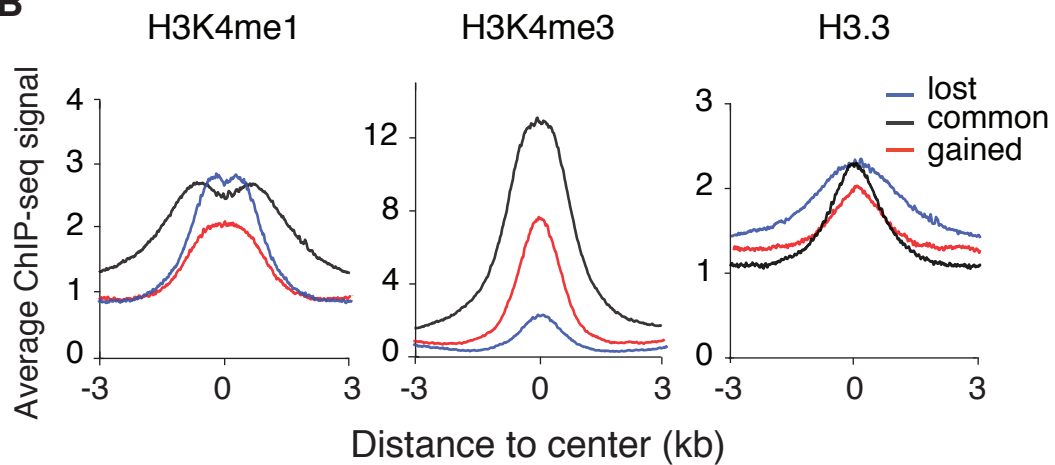**C**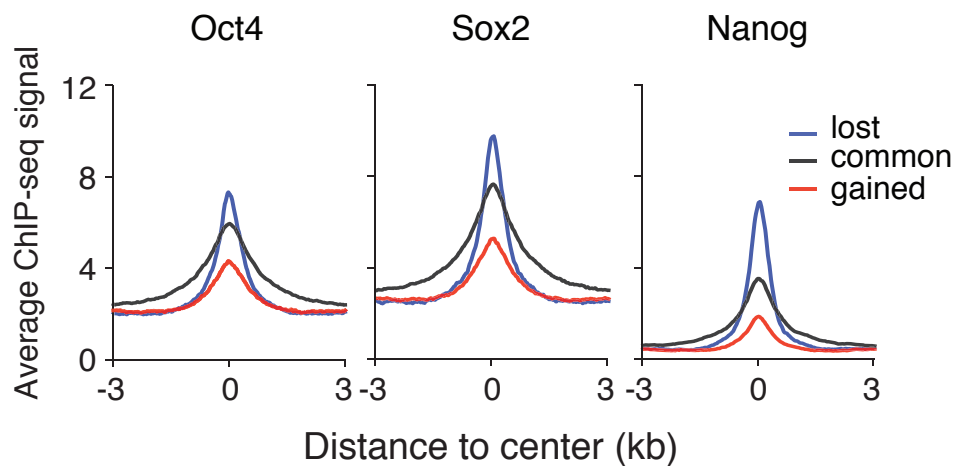**D**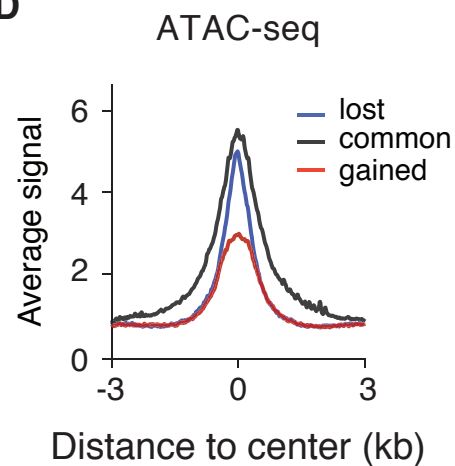

Supplement: Supplementary file 4 — Additional file 4: Figure S4. Chromatin features of p300-dependent enhancers in wild-type mESCs. (A) ChIP-seq average profiles of p300 [19] in wild-type mESCs at regions of H3K27ac enrichment that are lost, common and gained after p300 KD. (B) ChIP-seq average profiles of H3K4me1, H3K4me3 and H3.3 [19] in wild-type mESCs at regions of H3K27ac enrichment that are lost, common and gained after p300 KD. (C) ChIP-seq average profiles of Oct4, Nanog, Sox2 [29] in wild-type mESCs at regions of H3K27ac enrichment that are lost, common and gained after p300 KD. (D) ATAC-seq average profile in wild-type mESCs at regions of H3K27ac enrichment that are lost, common and gained after p300 KD. [file 12860_2020_296_MOESM4_ESM.pdf]

**A****Fig. 1A**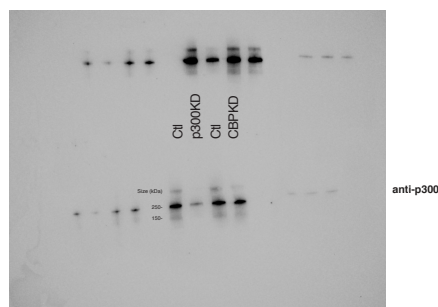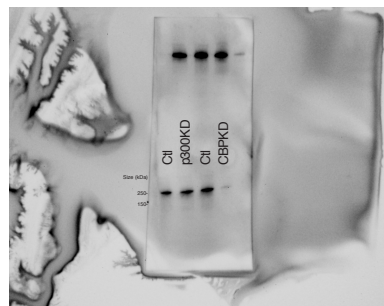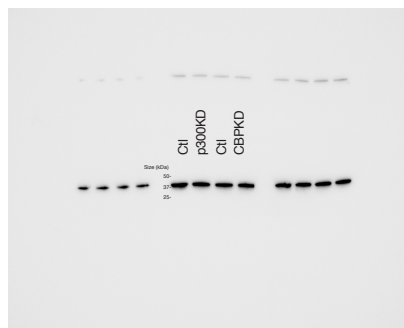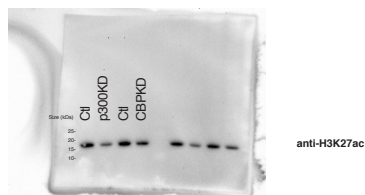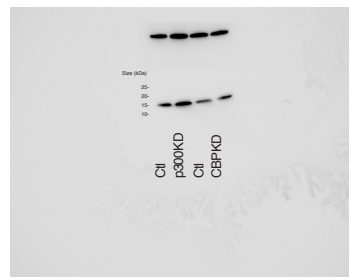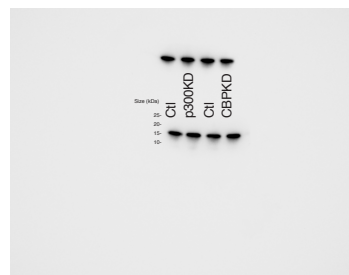**B****Suppl Fig. 1C**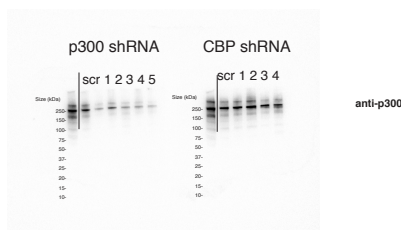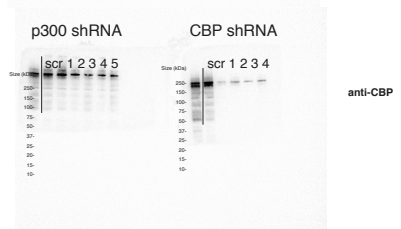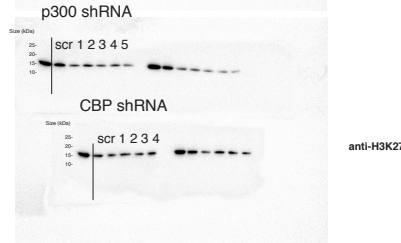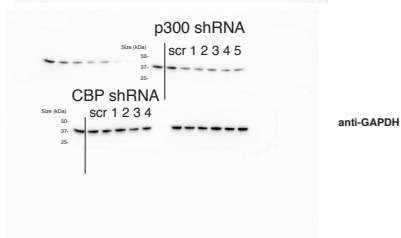**Suppl Fig. 1G**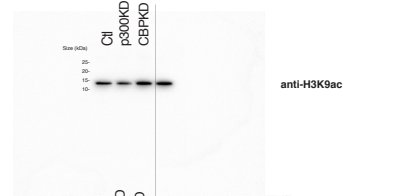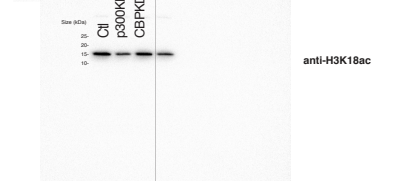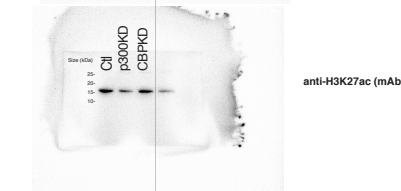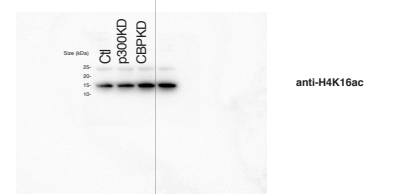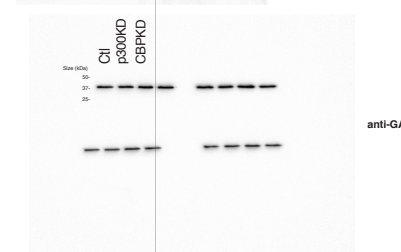**C****Suppl Fig. 3C**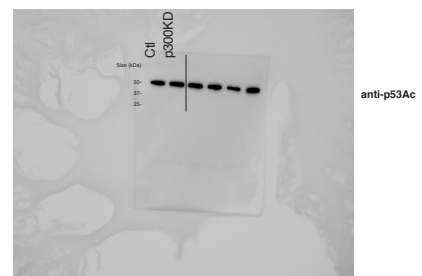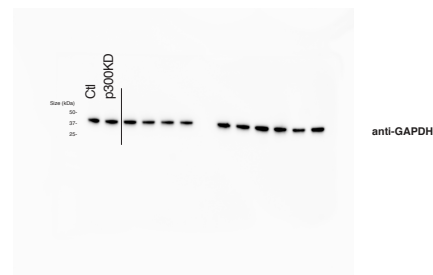**Suppl Fig. 3D**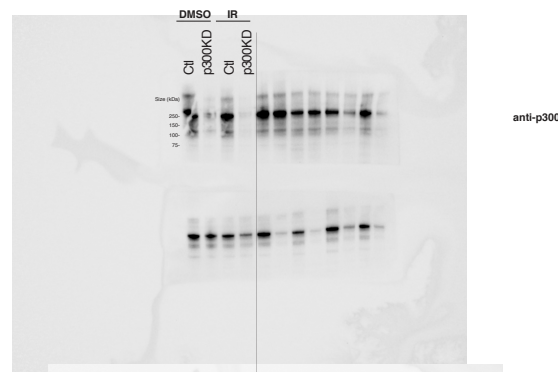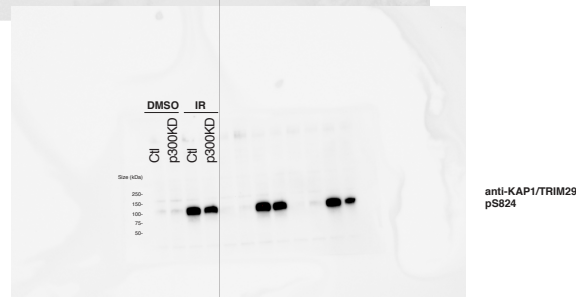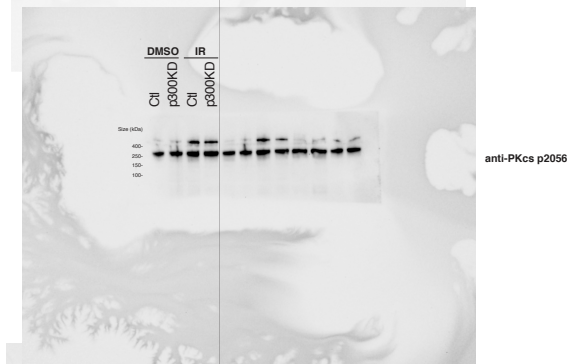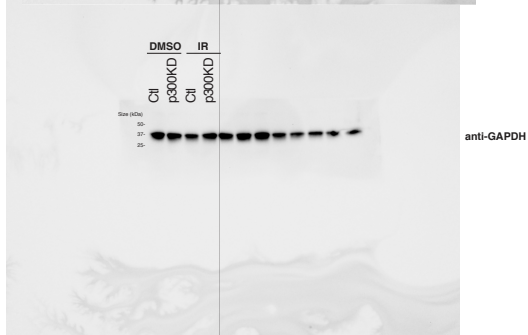

Supplement: Supplementary file 5 — Additional file 5: Figure S5. Raw immunoblots. (A) Western blots related to Fig. 1a. (B) Western blots related to Supplementary Figure 1C and 1G. (C) Western blots related to Supplementary Figure 3C and 3D. [file 12860_2020_296_MOESM5_ESM.pdf]
